# Venous Thromboembolism Prophylaxis in Plastic Surgery Patients Undergoing Facelift

**DOI:** 10.1093/asjof/ojac024

**Published:** 2022-04-12

**Authors:** Rohun Gupta, Jithin John, Monik Gupta, Kenneth Shaheen

**Affiliations:** Oakland University William Beaumont School of Medicine, Rochester, MI, USA; Oakland University William Beaumont School of Medicine, Rochester, MI, USA; The University of Toledo Health Science Campus, Toledo, OH, USA; Department of Plastic Surgery, Beaumont Health Systems, Royal Oak, MI, USA

## Abstract

**Background:**

In 2011, the American Society of Plastic Surgeons approved the Venous Thromboembolism (VTE) Task Force Report, which recommended the use of the Caprini scoring system, which has been adopted for VTE prophylaxis by most surgical societies in America.

**Objectives:**

The aim of this study is to investigate the incidence of deep vein thrombosis (DVT) and pulmonary embolism (PE) in patients undergoing facelifts at a single institution who did not undergo VTE chemoprophylaxis based on the Caprini scoring system.

**Methods:**

A retrospective chart review was conducted of patients who underwent facelift at a single institution. Patients were included if they were operated on between 2016 and 2021 by the lead surgeon and excluded if they received VTE prophylaxis. Descriptive statistics were conducted to analyze the collected data.

**Results:**

In total, 136 patients were isolated after chart review, and no patients were found to have had DVT or VTE. The average Caprini score was 5.625 and ranged from 3 to 10. There were 3 patients with evidence of postoperative hematoma (Caprini score = 5, 5, 7). The overall hematoma percentage was 2.21%.

**Conclusions:**

Based on the average Caprini score for the patients, all patients should have received VTE chemoprophylaxis. The authors found no VTE-related events in the patients without chemoprophylaxis. This study suggests that while the Caprini scoring system is a critical diagnostic tool for certain surgical procedures, it might not be optimal in predicting VTE in aesthetic patients undergoing surgical procedures.

**Level of Evidence: 4:**

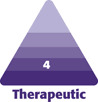

According to the 2020 plastic surgery report by the American Society of Plastic Surgeons (ASPS), the top 5 cosmetic procedures performed by plastic surgeons were nose reshaping, eyelid surgery, facelift, liposuction, and breast augmentation.^1^ Particularly, a total of 234,374 patients underwent rhytidectomy, with 21% of patients between the ages of 40 and 54 years and 64% of patients between the ages of 55 and 69 years.^1^ The primary purpose of rhytidectomy is to restore the contours, shape, and volume of the face to achieve a more youthful appearance. Full facelift techniques are categorized based on the plane of dissection. They include the superficial muscular aponeurotic system (SMAS) rhytidectomy, deep plane rhytidectomy, and the minimal access cranial suspension (MACS) lift.^2-4^ In recent years, nonsurgical and minimally invasive techniques have gained traction as adjuvants for facelifts. For instance, some physicians have begun to advocate for using hyaluronic acid fillers due to limited postoperative adverse events and minimal scarring.^5^ Other techniques include botulinum A neurotoxin, which has proven effective in reducing hyperfunctional lines throughout the face.^6^ Furthermore, surgical techniques such as SMAS continue to be the most popular method for rhytidectomy.^7^

Although there are numerous operative risks such as fever, hemorrhage, and infection, venous thromboembolism (VTE) is perhaps the topic of interest in plastic surgery.^8^ Due to a lack of evidence and consensus in the Plastic Surgery Literature, the ASPS formed the Venous Thromboembolism Task Force Report. The report introduced chemoprophylaxis recommendations in 2005.^9^ As part of the report, individualized VTE risk stratification and utilization of the 2005 Caprini score were recommended.^10^ The Caprini score at the time was broken down from 0-1 (low risk), 2 (moderate risk), 3-4 (high risk), and 5 + (highest risk).^11^ In 2013, the Caprini score was amended to include additional risk factors such as BMI above 40, smoking, diabetes requiring insulin, and length of surgery greater than 2 hours.^12,13^

However, since the Venous Thromboembolism Task Force Report was published, several studies have questioned the validity of the Caprini score for VTE prophylaxis.^14,15^ In fact, one study determined that the Caprini score has a 97% false-positivity rate.^15^ Another study found that risk assessment models, such as the Caprini score, have weak predictive accuracy for VTE.^16^ Due to insufficient evidence and heterogeneity, the study emphasized that there was no ability to recommend using any particular risk assessment model.^16^ The Caprini score was historically created for high-risk patients undergoing general surgery, orthopedics, or individuals who had a significant family history of diseases, such as hypercoagulability.^11^ Furthermore, when compared with cosmetic patients, patients undergoing general surgery tend to have vastly different health risks.^17^ A study by Winocour et al determined that the occurrence of VTE in cosmetic procedures was low, at an overall incidence of 0.09%, and that current ASPS guidelines for VTE prophylaxis are not explicitly designed for cosmetic patients.^18^ Additionally, the authors suggested that the scoring system does not take into account body region or the procedure that is being performed.^18,19^ Lastly, anticoagulation therapy has complications such as hemorrhage, thrombocytopenia, and osteoporosis.^20^ As a result, some surgeons report not administering chemoprophylaxis to their patients due to adverse events or less often due to lack of evidence specific to plastic surgery practices.^21^

In this retrospective cohort study, we present our experience with chemoprophylaxis in patients undergoing rhytidectomy compared with that endorsed by the ASPS. We investigated the incidence of deep vein thrombosis (DVT) and pulmonary embolism (PE) in these patients who did not undergo chemoprophylaxis. We expect to see no significant increase in the incidence of PE or DVT regardless of Caprini score in these patients.

## METHODS

A retrospective chart review was conducted on patients from the Beaumont Health System, MI, database once Institutional Review Board approval was acquired (Royal Oak, MI). Patients were included in this study if they underwent a rhytidectomy between January 1, 2016, and December 31, 2021, by the senior surgeon. Patients were excluded from this study if they received DVT prophylaxis. Written consent was provided, by which the patients agreed to the use and analysis of their data.

### Variables Collected

Data were accessed and collected through Epic (Verona, WI) electronic medical records (EMRs). Variables collected included Caprini score variables (age, sex, BMI, type of surgery, recent events in the last month, venous disease or clotting disorders, history of inflammatory bowel disease, acute myocardial infarction [MI], chronic obstructive pulmonary disease [COPD], present or previous malignancy, and other potential risk factors) along with associated procedures and operating room (OR) time.

### Outcomes

The primary outcome of this study is to document the incidence rate of symptomatic VTE found in the EMR of rhytidectomy patients who met our inclusion and exclusion criteria. Notes from plastic surgeons along with the additional medical staff were reviewed. In addition, the incidence rate of hematoma and other procedural adverse events were included as a secondary outcome. Lastly, we aimed to evaluate the efficacy of our prophylaxis technique to that of the technique endorsed by the ASPS.

### Statistical Analysis

Caprini scores were recorded for each patient as numerical values (0-17), and patients were stratified into groups based on the presence of complications. A 2 sample *t*-test was conducted on percentile data in order to evaluate for differences in rates of complications in female sex, mean age, BMI > 25, mean OR time (minutes), major surgeries 1 month before the procedure, smoking, diabetes, COPD/pulmonary issues, hypertension, cardiovascular disease, renal disease, cancer, history of DVT/PE, venous disease, clotting disorders, average Caprini scores, and the average number of procedures each patient underwent.

Patient data were recorded in Microsoft Excel (Redmond, WA), and tests for means, standard deviations, and significance levels were calculated utilizing the standard statistical formulas. *P*-values were considered statistically significant if *P* < 0.05.

## RESULTS

### Participants

From January 1, 2016, to December 31, 2021, 136 patients met our inclusion criteria, and all were operated on under the senior surgeon. No patients were excluded from this study, as all did not receive VTE prophylaxis. In addition, all patients underwent general anesthesia. All patients were encouraged to return to activities of daily living 48 hours postoperatively. Each patient followed up 1 day, 7 days, 6 weeks, and 3 months postoperatively (range, 1-90 days)

### Descriptive Data

The mean age was 65.05 ± 6.98 years, with the median age being 64 years old and the range being 49 to 80 years of age (Table 1). Overall, 95.59% of patients were females, 55 patients (40.44%) had a BMI > 25, 31 patients (22.79%) had hypertension, 4 patients (2.94%) had diabetes mellitus, and 77 patients (56.62%) had a history of smoking. The mean OR time was 290.61 ± 84.41 minutes, with the median OR time being 281.50 minutes.

**Table 1.** Summary of Patients’ Demographic Characteristics

Variable	Level	Response
Age of patient	N	136
	Mean (SD)	65.05 (6.98)
	Median	64
Sex of patient	Female (%)	95.59
	Male (%)	4.41
Body mass index (BMI) categories	BMI < 25	81
	BMI > 25	55
Time in operating room (minutes)	Mean (SD)	290.61 (84.41)
	Median	281.5
Hypertension	Not noted	105
	Present	31
Diabetes mellitus	Not noted	132
	Present	4
Tobacco use	Not noted	59
	Present	77
Caprini score	Mean (SD)	5.63 (1.46)
	Median	5

SD, standard deviation.

Thirty-one patients underwent a facelift, 41 patients with 1 additional procedure, 46 patients with 2 additional procedures, 16 patients with 3 additional procedures, and 2 patients with 4 additional procedures (Table 2). The most common additional procedures included blepharoplasty (n = 45), facial fat grafting (n = 28), and dermabrasion (n = 16). All 3 patients (2.21%) with postoperative complications suffered from a surgically managed hematoma (Table 3). There were no patients who had complications related to VTE. No patients received VTE prophylaxis (Table 4).

**Table 2.** Complication Rate by Procedure Type

Procedure type	No. of patients	Patients without complications	No. of patients with venous thromboembolism
Facelift	31	1	0
Facelift with 1 additional procedure	41	1	0
Facelift with 2 additional procedures	46	1	0
Facelift with 3 additional procedures	16	0	0
Facelift with 4 additional procedures	2	0	0
Total	136	3	0

**Table 3.** Univariate Analysis of Patient Cohort

Risk factor	Patients without complications	Patients with complications	*P* value (any complication)
Patients (n)	133	3	n/a
Mean age, y	64.88	72.67	0.0556
Female sex, %	96.24	66.67	0.0134
BMI > 25, %	41.35	0	n/a
Mean OR time, minutes	289.92	321.33	0.5259
Major surgery 1 month before, %	0	0	n/a
Smoking, %	56.39	66.67	0.7248
Diabetes, %	3.01	0	n/a
COPD/pulmonary, %	3.76	0	n/a
HTN, %	23.31	0	n/a
CAD, history of MI, or other cardiovascular disease, %	13.53	66.67	0.0099
Renal disease, %	4.51	0	n/a
Cancer, %	25.56	0	n/a
History of DVT/PE, %	1.5	33.33	0.0002
Venous disease or clotting disorder, %	8.27	33.33	0.132
Average Caprini score	5.63	5.67	0.9628
Females (n)	128	2	n/a
Average number of procedures	1.4	2	0.3107

CAD, coronary artery disease, COPD, chronic obstructive pulmonary disease; DVT, deep vein thrombosis; HTN, hypertension, MI, myocardial infarction; OR, operating room; PE, pulmonary embolism.

**Table 4.** Venous Thromboembolism Prophylaxis by Caprini Score

Caprini score	PPX used	No PPX	Fraction with PPX, %	Fraction with DVT, %	Fraction with hematoma, %
0-2	0	0	0	0	0
3-4	0	28	0	0	0
5-6	0	78	0	0	2.56
7-8	0	22	0	0	4.55
>8	0	8	0	0	0

DVT, deep vein thrombosis, PPX, prophylaxis.

When comparing patients with complications to those who did not have, patient age (*P* = 0.0556), mean OR time (*P* = 0.5259), smoking percentage (*P* = 0.7248), venous disease or clotting disorder (*P* = 0.132), and the average number of procedures (*P* = 0.3107) did not display statistical significance (Table 3). The average Caprini score for the group without complications was 5.63, whereas the Caprini score for the group with complications was 5.67 (*P* = 0.9628). Between the 2 groups, female sex (*P* = 0.0134), cardiovascular disease (*P* = 0.0099), and history of DVT or PE (*P* = 0.0002) displayed statistically significant results.

Twenty-eight patients were at low risk, 100 patients were at a moderate risk, and 8 were at high risk based on the Caprini scoring guidelines (Table 4). Patients at moderate risk for VTE had a hematoma rate of 3%, while high-risk patients had a hematoma rate of 0%. The 3 patients had a Caprini score of 5, 5, and 7.

## DISCUSSION

This study aims to present our institution’s experience with not chemoprophylaxing cosmetic patients undergoing facelifts compared with the current VTE prophylaxis guidelines endorsed by the ASPS. VTE, DVT, and PE are known surgical complications, and mortality associated with these conditions is procedure dependent.^22,23^ It is our conviction that facelift patients tend to have different risks compared with both general surgery and reconstructive plastic surgery patients. Often, facelift patients tend to have elective surgeries, and, as a result, surgeons often have the opportunity to indeed weigh the risks and benefits associated with a particular surgical procedure. Our study determined that the risk of bleeding and the potential to develop a hematoma was much higher than our observed risk of VTE in our patient population. Furthermore, it is critical to consider that surgical procedures for hematoma evacuation may result in further operative-related complications and the potential for patients to be dissatisfied with their surgical results.^24^ Based on the 2020 Plastic Surgery Statistics Report, we believe that our patient cohort is representative of cosmetic patients in the United States in terms of both gender and age.^1^

According to a 596 ASPS surgeons survey on VTE prophylaxis in cosmetic patients, 39% to 48% of plastic surgeons reported not administering chemoprophylaxis to their patients, with a concern for bleeding (84%) and a lack of evidence specific to plastic surgery practice (50%).^21^ Similarly, our patient population was not prophylaxed due to concerns of an increased hematoma formation rate. Utilizing the Caprini score in our patient cohort, 28 patients (20.58%) were determined to be at low risk, 100 patients (73.53%) were at moderate risk, and 8 patients (5.88%) were at high risk (Table 4). Based on the current Caprini guidelines, 108 of our patients (79.41%) should have received some form of VTE prophylaxis, whether that had been with low-dose heparin or low-molecular-weight heparin.^11^ Lastly, our postoperative protocol involved all patients receiving sequential compression devices along with immediate ambulation. Most procedures were treated as outpatient, but more complex cases were treated as inpatient and were discharged the following day.

According to task force members, there was limited evidence to make all-inclusive recommendations for VTE prophylaxis in plastic surgery patients and “accepted the premise that the surgical cases included in the orthopedic and general surgery literature search were similar enough in their anatomical location, degree of invasiveness, and patient population to make them comparable (from a venous thromboembolism risk perspective) to the following plastic surgery cases: major body contouring, abdominoplasty, major breast reconstruction, major lower extremity procedures, and major head/neck cancer procedures.” ^9^ Although our studied patient population underwent head/neck procedures, we would not characterize the surgical procedure as being “major.” Furthermore, we maintain the belief that patients who undergo rhytidectomy should be treated differently than other cosmetic patients who undergo significant procedures such as body contouring and reconstructive procedures, which have been demonstrated to have higher rates of VTE.^25^

A 2016 study by Winocour et al found that patients who underwent cosmetic procedures had a 0.09% rate of confirmed VTE (0.01% VTE risk for facial procedures) and that combined procedures led to significantly higher rates of VTE in comparison to single procedures.^18^ However, in our patient population, we found no statistically significant difference in the rate of VTE based on the number of procedures that were performed. As in our study, the study also found that diabetes and smoking were not significant risk factors for the rate of VTE.^18^ Our study is limited in assigning significance to data points due to the limited sample size of patients with complications, and, as a result, we cannot rule out type 1 errors. Lastly, the paper found that the VTE risk for facial procedures was 0.01%, which is similar to other documented studies.^18,26^

Hematoma is generally the most common complication of cosmetic procedures. Other common complications include seroma formation, wound dehiscence, scarring, blood loss, and complications from anesthesia.^27^ Previously published literature has indicated that the incidence of hematoma is between 1.8% and 9%.^28,29^ Another study by Matarasso et al found a postoperative hematoma rate of 4.4% in 12,325 patients.^30^ In our patient population, the hematoma rate was 2.21%, which is in accordance with other published literature. Our incidence of hematoma is on the lower end, which may be multifactorial. It may be due to not confining to the Caprini guidelines and not administering prophylaxis to our patients or due to our small sample size. When stratified by Caprini score and risk category, all 3 patients were noted to be of moderate risk for VTE with scores of 5, 5, and 7. BMI, OR time, and venous disease were not statistically significant factors that led to an increased rate of hematoma. In addition, the Caprini score for these patients was in the mean and standard deviation of all patients, and the value was not statistically significant. Two of our patients with hematoma formation did have a history of cardiovascular disease, which was found to be significant. Although hypertension has been a documented factor to increase the rate of hematoma formation, we did not experience this in our patient population. This is consistent with another study by Rees et al.^29,31^ Lastly, there have been studies that have determined that VTE prophylaxis does indeed lead to increased rates of bleeding that requires reoperation.^32^

Current literature is conflicted on plastic surgeons’ stance when operating on cosmetic patients. Particularly for facelifts, a study by Broughton et al determined that only 48.7% of providers administered VTE prophylaxis.^33^ A study by Abboushi et al recommends the utilization of VTE prophylaxis regimen for cosmetic procedures, even “presumed low-risk procedures such as facelifts.” ^34^ Although it may be argued that prophylaxing patients leads to decreased risk of VTE, authors and providers must consider the potential burden this may cause on patients who do not necessitate prophylaxis. Other studies have supported the recommendation of routine prophylaxis for all types of surgical procedures, including reconstructive and aesthetic patients.^35^ These studies are limited by pooling all plastic surgery patients together and not stratifying patients based on the risks of each procedure, which would make findings more reliable.^36^ No surgeon willingly exposes his patients to the risk of VTE; however, we feel this and other studies similar to this will help elucidate which patient populations benefit from chemoprophylaxis.

Our study does contain limitations that may be seen as statistically significant. Our dataset has a small sample size, particularly in the subset of patients who experienced complications (*n* = 3). This may be seen as a notable limitation in conducting statistical analysis. However, it is imperative to consider that with our protocol of not providing prophylaxis to the patients, there were only 3 patients who experienced complications. In addition, due to differences in EMR systems between our institution and other nearby hospitals, it is possible that patients may have experienced complications and chosen not to return to our institution. As a result, any potential complications that may have occurred, would not show up in our EMR charting system. Thus, there may be the possibility that there were additional complications. Also, due to the utilization of Epic, there may be the possibility that a patient’s risk factors were not entirely present in the chart. As a result, the authors utilized a patient’s preoperative report, problem list, and provider notes to mitigate potential errors in calculating a patient’s Caprini score.

Furthermore, all patients came from a single surgeon at a single facility, and, as a result, the potential for skill bias cannot be discarded. Lastly, as a retrospective review, it is critical to note that a patient’s current Caprini score may reflect what may have been documented at the time of the procedure. Thus, it may be helpful to analyze how a patient’s Caprini score changes over time in future studies and how this may reflect VTE prophylaxis.

## CONCLUSIONS

The Caprini scoring system is a critical diagnostic tool utilized in patients undergoing certain surgical procedures. However, the scoring system should not be applied to all cosmetic patients. The score has clearly established that most aesthetic patients are at low risk of VTE, and the majority of these patients require noninvasive methods of thromboembolism prevention.^37^ In our study population, after careful data analysis, we determined that it is not unreasonable to question the broad utilization of the Caprini score in cosmetic patients, particularly those undergoing facelifts. Furthermore, additional studies are required in order to elucidate potential risk factors in cosmetic patients that may affect the quality of care and satisfaction of patients. Doing so may help create a scoring system that is truly dedicated to cosmetic patients.
